# Assessing Global Ionosphere TEC Maps with Satellite Altimetry and Ionospheric Radio Occultation Observations

**DOI:** 10.3390/s19245489

**Published:** 2019-12-12

**Authors:** Wei Li, Longqiang Huang, Shaocheng Zhang, Yanju Chai

**Affiliations:** 1 School of Geography and Information Engineering, China University of Geosciences, Wuhan 430074, China; weili@cug.edu.cn (W.L.); cug.hlq@cug.edu.cn (L.H.); 2 State Key Laboratory of Geodesy and Earth’s Dynamics, Institute of Geodesy and Geophysics, Chinese Academy of Sciences, Wuhan 430077, China; cyjigg@whigg.ac.cn; 3 Guangzhou Urban Planning & Design Survey Research Institute, Guangzhou 510060, China

**Keywords:** global ionosphere map, total electron content, satellite altimetry, ionospheric radio occultation, Jason-2, COSMIC

## Abstract

As global navigation satellite system (GNSS)stations are sparsely distributed in oceanic area, oceanic areas usually have lower precision than continental areas on a global ionosphere maps (GIM). On the other hand, space-borne observations like satellite altimetry (SA) and ionospheric radio occultation (IRO) have substantial dual-frequency observations in oceanic areas, which could be used for total electron content (TEC) retrieval. In this paper, the Jason-2 SA and Constellation Observing System for Meteorology, Ionosphere, and Climate (COSMIC) IRO products were used to assess the precision of IGS GIM products. Both the systematic biases and scaling factors between the international GNSS service (IGS) GIM TEC and space-borne TEC were calculated, and the statistical results show that the biases and the scaling factors obviously vary under different temporal-spatial conditions. This analysis shows that these differences are variable with diurnal and latitude factors, that is, the differences in biases during the day time are higher than those during the night time, and larger biases are experienced at lower latitude areas than at high latitude areas. The results also show that in the southern hemisphere middle-high latitude area and some other central oceanic areas, the space-borne TEC values are even higher than GIM TEC values. As the precision of space-borne TEC should be evenly distributed around different areas on Earth, it can be explain that the TEC in these areas is undervalued by the current GIM model, and the space-borne SA and IRO techniques could be used as complementary observations to improve the accuracy and reliability of TEC values in these areas.

## 1. Introduction

The ionospheric total electron content (TEC) is an important parameter for space weather research; the global ionosphere map (GIM) from the International GNSS (global navigation satellite system) Service (IGS) analysis center is one of the most famous ionospheric TEC products. As ionosphere TEC grids were generated based on the GNSS observations from hundreds of ground-based IGS stations, the precision of GNSS GIM products inevitably depends on the distribution of the ground IGS stations. This leads to unrealistic zero or negative TEC values in the first stage GIM computation [1,2]. This problem is solved with the interpolation method and background constraint conditions. Mannucci et al. (1998) used TEC predictions from a climatological model as simulation data to cover significant measurements gaps [3]. Orus et al. (2005) improved vertical TEC (VTEC) maps using the Kriging interpolation algorithm, and the new Polytechnic University of Catalonia (UPC) kriging GIM has a lower RMS of about 16% and 2%, respectively, in the calculated slant TEC (STEC) than the original UPC GIM and IGS GIM in the self-consistency test [4]. Mautz et al. (2005) presented the B-spline wavelets method to represent the spatial and temporal variations of GIM and to solve the problems in ionospheric modeling due to data gaps [5]. The inequality-constrained least square (ICLS) method was proposed by Zhang et al. (2013) to eliminate nonphysical negative values in ionosphere associate analysis centers (IAACs) GIM products [2]. Li et al. (2015) proposed an approach named Spherical Harmonic plus generalized Trigonometric Series functions (SHPTS) to improve the accuracy and resolution of GIM; the accuracy of the SHPTS-based GIM could achieve 2-6 TECU over the area without GNSS measurements [6]. Wang et al. (2016) used priori VTEC values calculated from the IRI 2012 model to replace the grid points of GIM with negative VTEC values [7]. However, such interpolation and background constraint methods could not fully reflect the real situation of the ionosphere in ocean areas due to a lack of GNSS observations. 

As satellite-based observations are not constrained by the Earth’s surface patterns, researchers are trying to use satellite-based dual-frequency observations to complement the insufficient GNSS observations over ocean areas. Todorova et al. (2007) and Schmidt et al. (2007) combined GNSS and satellite altimeter (SA) observations to build a global and regional ionosphere TEC model, respectively [8,9]. Hernandez-Pajares et al. (2009) evaluated the precision of the GIM TEC by using SA data [10], and Alizadeh et al. (2011) used ground-based GNSS, SA and global positioning system (GPS) ionospheric radio occultation (IRO) observations to model the GIM by weighted least squares estimation [11]. Their results indicate that the precision and reliability of the GIM have been improved significantly. Dettmering et al. (2011) computed regional VTEC models based on the data from IRI 2007, terrestrial GPS observations, dual-frequency SA, IRO, and Very Long Baseline Interferometry (VLBI) [12]. Chen et al. (2017) used multi-source data (including ground-based GNSS, SA, IRO, and doppler orbitography and radiopositioning integrated by satellite (DORIS)) with different weights determined by Helmert variance component estimation to build GIM products [13]. Hu et al. (2019) proposed an approach named Helmert-aided fast-weighted total least squares to improve the accuracy of multi-source data GIM modeling [14]. 

However, space-borne observations are limited below the low earth orbit (LEO) satellite orbits, which could not reflect TEC values for the whole ionosphere. Scharroo et al. (2010) used SA TEC data over six-month period to compare with Jet Propulsion Laboratory (JPL, Pasadena, CA, USA) GIM TEC and analyzed the scaling factors between SA TEC and JPL GIM TEC [15]. In their results, the scaling factors were considered as constants without variance over time, location, and solar activity status; the scaling factors were defined as 0.925 for TOPEX (TOPography EXperiment), 0.915 for Jason-1, and 0.856 for Envisat. Yizengaw et al. (2008) used Jason-1 TEC to estimate the relative contribution of the plasmaspheric TEC to GPS TEC [16], and Chen et al. (2015) used Constellation Observing System for Meteorology, Ionosphere, and Climate (COSMIC) TEC to estimate the contribution of plasmaspheric TEC to GPS TEC [17]. Both of these studies’ results show that the contribution exhibits a significant diurnal variation, which is also affected by latitude and season. However, both studies directly treated the SA or IRO TEC values, which were greater than IGS GIM TEC as outliers and discarded them in the data processing. Liu et al. (2018) noted that TOPEX/Jason TEC values were greater than GNSS-derived TEC while they extracted plasmaspheric TEC and explained the phenomenon as the systematic biases from TOPEX/Jason, which led to an overestimation of TOPEX/Jason VTEC [18]. 

In this research, we evaluated the GNSS GIM TEC with Jason-2 SA and COSMIC IRO TEC and investigated topside TEC variations with local time (LT), latitude, longitude, and season. The global distribution of the difference between space-borne TEC and IGS TEC was plotted to assess anomalous regions and determine possible reasons for such anomalies. This paper is organized as follows: After a brief introduction of the research background in Section 1, the principles of the ground-based GNSS ionospheric TEC retrieval methods and their limitations are discussed in Section 2; then, the space-borne ionosphere TEC retrieval techniques and datasets utilized in this study are introduced in Section 3; Section 4 presents some evaluation results and discussions, and Section 5 offers the conclusion and outlook for our study.

## 2. Principles of Ground Based GNSS TEC Retrieval 

The massive free electrons of the ionosphere could affect GNSS signals and result in measurement biases at different frequency signals. Based on this characteristic, the STEC through the signal path could be retrieved from dual-frequency GNSS code and phase observations. The geometry-free (GF) code and phase formulas are denoted as follows [19]:

(1) $$P_{1}-P_{2}=\frac{40.3\left(f_{2}^{2}-f_{1}^{2}\right)}{f_{1}^{2}f_{2}^{2}}STEC+B_{r}-B^{s}+\varepsilon_{p}\;\phi_{2}-\phi_{1}=\frac{40.3\left(f_{2}^{2}-f_{1}^{2}\right)}{f_{1}^{2}f_{2}^{2}}STEC+a_{\mathrm{GF}}^{s}+\varepsilon_{\phi }$$

where $f_{1}$ and $f_{2}$ are the GNSS frequencies, $P_{1}$ and $P_{2}$ are code measurements, $\phi_{1}$ and $\phi_{2}$ are phase measurements, $B_{r}$ and $B^{s}$ are the Differential Code Biases (DCB) of receiver and satellite respectively, $a_{\mathrm{GF}}^{s}$ is the float ambiguities of the GF measurements, and $\varepsilon_{p}$ and $\varepsilon_{\phi }$ are code and phase measurement noise. All the parameters, expect for the GNSS frequencies, are denoted by meters. By averaging the GF code and phase measurements for every continuous arc, the float ambiguities of the GF measurements are estimated and subtracted from the GF phase measurements.

In the Single-Layer Model (SLM) assumption, the ionosphere is considered as a thin shell that contains most ionospheric electron density [20,21], with an altitude generally assumed to be 450 km. The intersection of the shell and the signal path is named as ionospheric pierce point (IPP). In order to model the ionosphere conveniently, the STEC is usually mapped to the VTEC at the IPP, as [19]:

(2) $$STEC=\frac{1}{cos\;z^{\prime }}VTEC$$

where $z^{\prime }=arcsin\frac{R_{E}sinz}{R_{E}+H}$, H=450 km, $R_{E}$ is the Earth’s radius, and z is the zenith angle of the receiver.

Based on the dual-frequency GNSS observations from global IGS stations, the spherical harmonics function and trigonometric series function are used to build GIM products [22,23,24]. Since GPS IPPs cover most of the continental area but are sparse in ocean and polar regions, the precision of GIM TEC is low in the data missing areas.

## 3. Space-Based Ionospheric TEC Retrieval and Datasets

The SA technique has no limitation on the ground stations and is best applicable over oceans [12,25]. The advantage of the radio occultation technique is that the data are globally distributed and not limited to continental regions [26]. The TEC retrieval methods for the two space-borne observations are summarized first. The datasets utilized in this study are then introduced. Since 2014 is a high solar active year, two different space-based TEC products from 2014 are utilized to evaluate the IGS GIM products on ocean areas. Each dataset is divided into four groups according to seasonal factors [27]: M-Equinox (March, April, and May), J-Solstice (June, July, and August), S-Equinox (September, October, and November) and D-Solstice (December, January, and February).

### 3.1. Satellite Altimetry TEC Retrieval

The on-orbit SA missions with dual-frequency are currently executed by Jason-2/3, HY-2A, and Sentinel-3. The space-borne dual-frequency (Ku band: 13.75 GHz, C band: 5.3 GHz) altimeter transmits signals vertically to earth and receives the echo from the sea surface to derive the vertical range between the satellite and the sea surface based on the travel time. According to Equation (3), VTEC below the satellite’s orbital height could be directly calculated by using dual-frequency altimeter measurements [28].

(3) $$h_{Ku}-h_{C}=-\frac{40.3\left(f_{C}^{2}-f_{Ku}^{2}\right)}{f_{Ku}^{2}f_{C}^{2}}VTEC+\left(S_{Ku}-S_{C}\right)+\left(B_{Ku}-B_{C}\right)$$

where $f_{Ku}$ = 13.75 GHz and $f_{C}$ = 5.3 GHz, and $h_{Ku}$ and $h_{C}$ are ranges of the Ku and C band signals, respectively. $S_{Ku}$ and $S_{C}$ are the sea state bias correction in the Ku and C bands, respectively. $B_{Ku}$ and $B_{C}$ are the instrumental errors correction on the Ku and C bands, respectively.

### 3.2. Jason-2 Satellite Altimetry Data

The Jason-2 altimeter measurements from the final GDR (Geophysical Data Record) product of the Centre National d’Etudes Spatiales (CNES) are utilized in the following evaluation (ftp://avisoftp.cnes.fr/AVISO/pub/jason-2/gdr_d). The final GDR products provide the ranges from satellite to sea surface ($h_{Ku}$ and $h_{C}$) and the corresponding sea state bias corrections ($S_{Ku}$ and $S_{C}$) measured in different frequency bands, as well as the ranges with the modeled instrumental errors corrections ($B_{Ku}$ and $B_{C}$). However, instrumental noise errors still exist in the range measurements. To reduce the random observational noise, the handbook of Jason-2 products from CNES recommends to apply a smoothing filter in the processing, with the filter windows set as 20–25 along with 25–35 successive measurements for local time 06:00–24:00 and 00:00–06:00, respectively [28]. After introducing the above measurements and corrections into Equation (3), the VTEC are retrieved.

**Figure 1:**
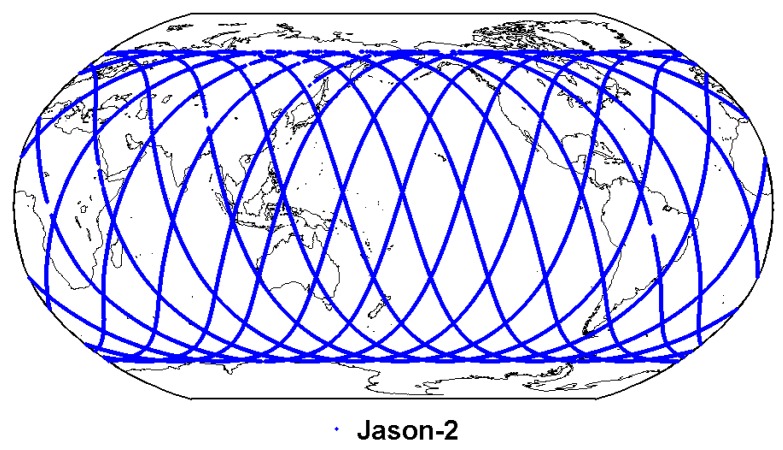
The footprints distribution of Jason-2 altimeter data on 1 January 2014.

The Jason-2 satellite operates at an orbital altitude of 1336 km, with an inclination of 66°. As shown in Figure 1, the footprints of Jason-2 cover most ocean areas within 66° S~66° N. Therefore, the Jason-2 altimeter TEC could be effectively used to evaluate IGS GIM products over ocean areas that lack ground-based ionosphere measurements [28]. 

### 3.3. Ionospheric Radio Occultation TEC Retrieval

The main operating GPS IRO missions are executed by the COSMIC, CHAMP, GRACE, and FY-3C, while the CHAMP and GRACE satellites were decommissioned in 2008 and 2017, respectively (https://cdaac-www.cosmic.ucar.edu/cdaac/products.html). When the GPS occultation events happen, the on-board GPS occultation receiver receives L1 and L2 signals from the GPS satellites and provides dual-frequency phase measurements. Utilizing the dual-frequency phase measurements and the precise positions of the GPS and LEO satellites, the relative TEC of the signal propagation path could be calculated. Under the assumption of spherical symmetry, the ionospheric electron density of the tangent points could be derived by using Abel transform, and then, the TEC values could be determined by integration of the electron density profiles [29,30].

However, the COSMIC IRO TEC inversion experiences several important error sources. Firstly, the Abel transform method is under the assumption that the electron density in the ionosphere is spherically symmetric. This assumption is not satisfied, especially in the active period of the ionosphere, as well as at low latitudes where there are strong horizontal gradients of ionospheric electron density. Therefore, the assumption of spherical symmetry may introduce significant systematic errors [31]. Secondly, the electron density profile in occultation events is not a straight line but a curve, and therefore, it would bring in certain errors when mapping into VTEC. In addition, the presence of ionospheric density irregularities will cause scintillation and multipath effects, which often create some small-scale fluctuations of random electron density profiles [32].

### 3.4. COSMIC Ionospheric Radio Occultation Data

In this study, the COSMIC VTEC values from the “ionPrf” product of the University Corporation for Atmospheric Research (UCAR, Boulder, CO, USA) COSMIC Data Analysis and Archival Center (CDAAC) are used for the following investigation, and the TEC values are calculated by integration of the electron density profile and mapping to vertical direction. The derived range is from 80 km to the COSMIC satellite orbital height (about 800 km). As mentioned above, some ionospheric electron density profiles are not reliable due to the presence of ionospheric irregularities and occultation point paths over bending. Hence, a quality control approach to discard the questionable electron density profiles is necessary [33]. In this research, the profiles in the following cases have been removed: (1) the profiles that have obvious bending on the occultation point trace; in the data pre-processing, distance of the straight-line connecting the top and bottom tangent points (D1) and distance of the perpendicular line from F2 peak point to the straight-line (D2) are calculated. If the ratio of D2/D1 is larger than 10%, the profile is considered as obvious bending on the occultation trace and needs to be removed. (2) Since the retrieved electron density of some profiles provided by CDAAC would be negative in the bottom layer of the ionosphere, the profiles whose electron density above 120 km contain negative values are also removed.

**Figure 2:**
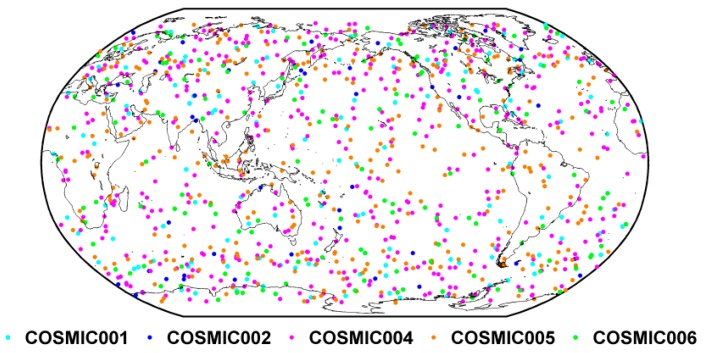
The distribution of COSMIC (Constellation Observing System for Meteorology, Ionosphere, and Climate) occultation event on 1 January 2014. Different colors represent different COSMIC satellites.

Figure 2 shows the distribution of COSMIC occultation events on 1 January 2014. In Figure 2, generally, the occultation events are evenly distributed in both ocean and continental areas, and in terms of density distribution, the density of the occultation events at high latitudes is higher than those in low latitude areas.

## 4. Result Analysis 

The IGS GIM products in IONEX (IONospheric map Exchange) format provide the VTEC at grid points with a spatial resolution of 5° × 2.5° (longitude × latitude) at an interval of 2 h. The SA VTEC below orbital height could be directly calculated at the Jason-2′s footprints from the final GDR products, and the geographical location of footprints and corresponding measurement time (local time) are stored for the following evaluation as well. For the COSMIC, the “ionPrf” product from CDAAC provides the VTEC, as well as the longitude, latitude and altitude of the peak electron density in the ionospheric F2 layer; among them, the altitude of the peak electron density in the ionospheric F2 layer is defined as hmF2. The SA and COSMIC VTEC were compared with the corresponding values provided by the IGS GIM products by applying accurate temporal (in local time) and spatial (longitude and latitude) interpolation algorithm as suggested in [34].

### 4.1. Evaluation of IGS GIM TEC with Jason-2 SA TEC

**Figure 3:**
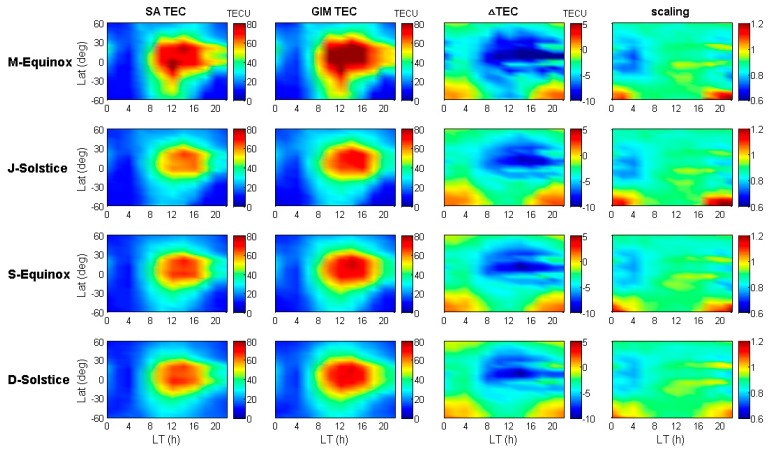
Jason-2 and International GNSS Service (IGS) global ionosphere map (GIM) total electron content (TEC) variations for the four seasons of 2014.

Figure 3 demonstrates the Jason-2 and GIM TEC variations with geographical latitude and local time for the above defined four seasons of 2014. The x-axis and y-axis in each subplot are the local time and geographical latitude, respectively. The four defined seasons (M-Equinox, J-Solstice, S-Equinox and D-Solstice) are indicated in Figure 3 from the top to bottom row. Firstly, the ionospheric VTEC is retrieved from the Jason-2 dual-frequency observations for each season, and the latitude and local time of the footprints are recorded with the corresponding VTEC values. Then, the SA TEC at each footprint is indicated in the first column; Secondly, the VTEC of ground-based GPS is interpolated into the location of footprint by using the IGS GIM products; the result of this step is shown in the second column. The third column shows the bias between Jason-2 and GIM TEC ($\Delta TEC=TEC_{SA}-TEC_{GIM}$), and the fourth column shows the scaling factor between Jason-2 and GIM TEC ($scaling=TEC_{SA}/TEC_{GIM}$).

The first and second column of Figure 3 shows the Jason-2 and IGS GIM TEC variations with geographical latitude and local time, respectively. The third column clearly shows that the IGS GIM TEC values are generally greater than those of Jason-2 TEC because of the existing topside ionospheric TEC and plasmaspheric TEC above the Jason-2 orbit. Moreover, the differences were obviously higher during daytime (06:00–18:00) than nighttime (18:00–06:00). Compared with column one, the greater the Jason-2 TEC values, the greater the difference between Jason-2 and GIM TEC, where the maximum difference could reach around −10 TECU in low latitudes during the daytime. However, there are special cases in mid-high latitudes of the southern hemisphere from 16:00 to 06:00 (LT), where the GIM TEC results are underestimated about 2~3 TECU than the Jason-2 TEC results. Theoretically, this result is abnormal, as the orbital altitude of Jason-2 is much lower than that of the GNSS satellite. Considering that the footprint distribution of Jason-2 is even, the retrieval accuracy of VTEC from the Jason-2 measurements is not affected by spatial distribution. These special cases are defined as GIM TEC underestimated phenomenon in this study.

As shown in the fourth column of Figure 3, the diurnal scaling variations show a pattern, with 0.7~0.9 at night and 0.9~1.0 on daytime. This pattern means that the percentage of the topside TEC at night is higher than that during the daytime, even though the nighttime ionospheric electron content is much lower than that during the daytime. In the daytime, the ionosphere electrons increase rapidly with an increase in solar radiation and diffuse up into the topside, so the topside electron content increases. During nighttime, the ions and free electrons recombine, which makes the ionosphere electrons decrease sharply, and the topside electrons also diffuse back to the bottom side, thereby decreasing the topside electron content. The rate of ionosphere electron diffusion is much slower than the rate of their increase or decrease, resulting in a higher contribution of topside TEC in the nighttime than in the daytime [17]. However, in the middle latitude southern hemisphere, the underestimated phenomenon of GIM appears at night, so the corresponding scaling values are greater than 1.

**Figure 4:**
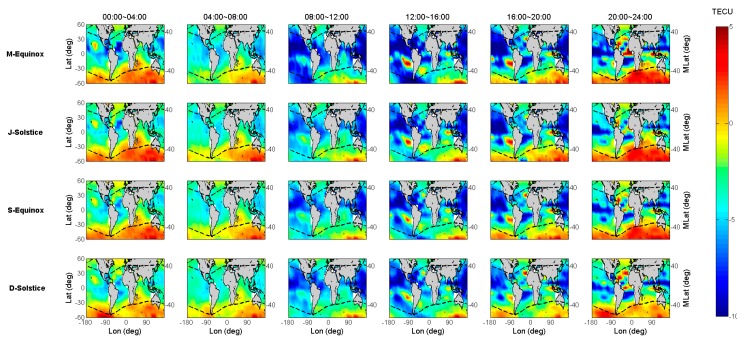
The global distribution of biases between Jason-2 and IGS GIM TEC for the four seasons of 2014.

**Figure 5:**
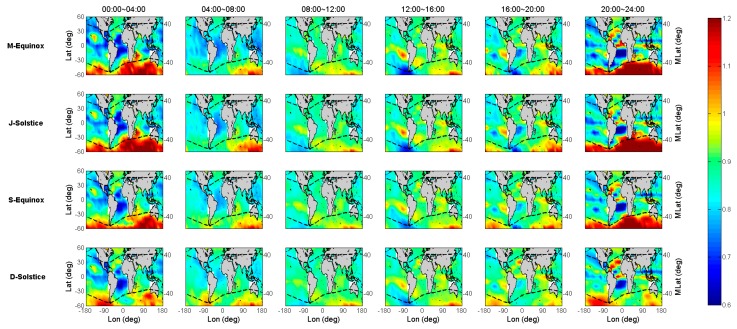
The global distribution of scaling between Jason-2 and IGS GIM TEC for the four seasons of 2014.

As seen in Figure 3, the variation tendency of Jason-2 SA TEC is similar to that of IGS GIM TEC, and the former values are generally lower than the latter ones because the height of the LEO satellite orbit is inside the ionosphere, except for some areas, such as the mid-high latitudes of the southern hemisphere. Since the results shown in Figure 3 only take the TEC variations into account with geographical latitude and local time, the detailed location where the anomaly appeared could not be identified. In order to look into the distribution of GIM TEC underestimated phenomenon, we also plotted the global distribution of the bias and scaling by taking into account the impact of longitudinal differences. The results of the bias and scaling with spatial and temporal variations are shown in Figure 4 and Figure 5, respectively.

In Figure 4 and Figure 5, the seasonal results are indicated in the similar way as Figure 3, where the x-axis is replaced by geographical longitude, and the y-axis still denotes geographical latitude; the panels at different columns represent different local time periods with a duration of 4 h, and the black dashed line indicates the geomagnetic latitude line at ±40°. After considering the longitudinal differences, the IGS GIM TEC underestimated phenomenon were mainly found to be distributed over sea areas near the geomagnetic equator, and the mid-high geomagnetic latitudes exceeded 40° of the southern hemisphere at a certain time 20–04 (LT). Based on the different column panels, different local time periods clearly have different anomaly distributions. From 08:00 to 16:00 (LT) as shown in the third and fourth columns, the underestimated GIM TEC are scattered in the South Indian Ocean, the South West Pacific Ocean, and the west coast of South America. In general, the area of the underestimated phenomenon distribution during this period is not obvious compared with that of the other period. However, when the night falls, the underestimated areas in the southern hemisphere begin extending to the most mid-high geomagnetic latitude oceans, and the largest underestimated area range appears at midnight. Compared with Figure 3, this phenomenon lasts until 06:00 (LT). For seasonal variations, during the D-Solstice season, the underestimated phenomenon at mid-high geomagnetic latitudes seems to be not very obvious compared to those of the other defined seasons. In summary, the underestimated IGS GIM TEC are mainly distributed in the mid-high geomagnetic latitudes of the southern hemisphere and nearby the geomagnetic equator where ionosphere anomalies occur frequently. It is shown that the underestimated phenomenon is also experienced more in the nighttime than in the daytime, as well as more often in the M-Equinox, J-Solstice, and S-Equinox seasons than in the D-Solstice season. The biases and scaling factors almost reach 5 TECU and 1.2, respectively, in the region exceeding 40 geomagnetic latitudes of the southern hemisphere at a certain time 20–04 (LT). 

Since the GIM products are generated based on a Sun-fixed frame, the center point longitude takes the sun-fixed longitude as the parameter, which means the ‘mid-night’ areas are the opposite/boundary areas at the center of the sphere-harmonic model. If there are enough observations in those ‘mid-night’ areas, the accuracy could be promising, e.g., the north hemisphere. However, most of the south hemisphere is filled with oceans, which do not have enough observations. Consequently, their TEC values cannot be correctly modelled with the sphere-harmonic function. This factor may result in some ‘holes’ with negative values in these areas. In this case, a second step will need to use an optimum spatial-temporal interpolation technique to extend the estimates to cover the entire Ionosphere [1].

As mentioned above, insufficient ground-based GNSS observations in such areas might result in a low precision of extrapolated TEC values, and eventually underestimated GIM TEC appear. For the well-defined diurnal variations of the underestimated phenomenon, the approach of IGS GIM modeling by using a sun fixed coordinate might cause these diurnal variations because of the boundary effect of the function model. Therefore, considering sufficient observations and no environmental limitation of SA, the precision of VTEC from Jason-2 dual-frequency observations can maintain stably. Thus, under the premise of ensuring Jason-2 precision, the bias and scaling results indicate that the real ionospheric environments in many regions could not be well reflected by IGS GIM.

### 4.2. Evaluation of IGS GIM TEC with COSMIC IRO TEC

**Figure 6:**
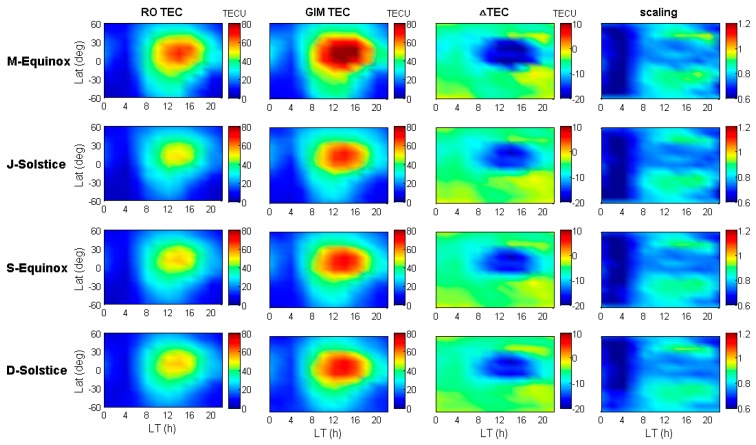
COSMIC and IGS GIM TEC variations for the four seasons of 2014.

The COSMIC and IGS GIM TEC variations are indicated in the first and second columns of Figure 6 with geographical latitude and local time for the four seasons of 2014. Since the number of COSMIC observations in 2014 is much less than that of Jason-2, the results of Figure 6 cannot demonstrate greater TEC variation details, especially in M-Equinox season.

The third column of Figure 6 shows the bias results between COSMIC and IGS GIM TEC ($\Delta TEC=TEC_{COSMIC}-TEC_{GIM}$). It shows that the difference between these two TEC results (ΔTEC) in the low latitudes of the northern hemisphere and near the equator is more obvious than those in other regions, and the maximum difference could decrease to –20 TECU between 10:00 and 18:00 (LT) in all seasons. For the other latitude regions apart from above-mentioned region, during the same period, the higher the latitude, the smaller the difference. The ΔTEC is between –5 and 0 TECU in the high latitude area, while it is between –10 and –5 TECU in other areas. However, in some mid-latitude regions at 16:00–22:00 (LT), the bias between COSMIC and IGS GIM TEC is close to zero.

The fourth column of Figure 6 shows the scaling factors between COSMIC and IGS GIM TEC ($scaling=TEC_{COSMIC}/TEC_{GIM}$). As seen in this column, there is a diurnal scaling variation with well-defined boundary at 08:00 (LT) in all seasons. From 00:00 to 08:00 (LT), the distribution of the scaling does not follow the regularities of the latitude variation, and the values are basically maintained around 0.6. After 08:00, the distribution of the scaling begins to show north-south symmetry with 0.85~0.95 around the 40° latitudes and 0.75~0.8 around the low latitudes.

**Figure 7:**
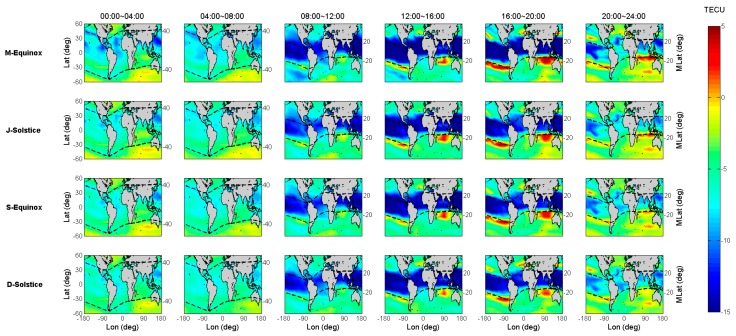
The global distribution of biases between COSMIC and IGS GIM TEC for the four seasons of 2014.

**Figure 8:**
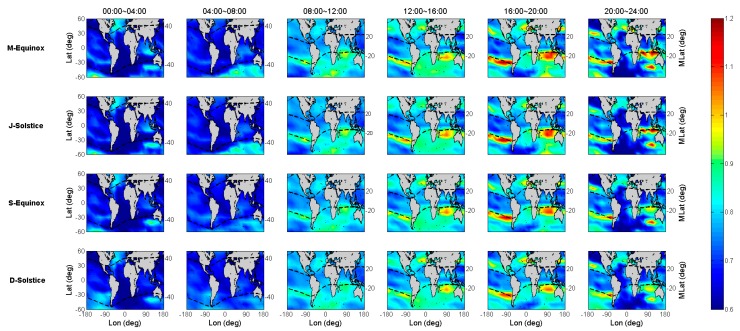
The global distribution of scaling between COSMIC and IGS GIM TEC for the four seasons of 2014.

To obtain more detailed distribution information for the bias and scaling factors between COSMIC and GIM TEC, Figure 7 and Figure 8 are plotted to show the bias and scaling variations with spatial and temporal, respectively.

In Figure 7, the bias of COSMIC and IGS GIM TEC in the equator between 08:00 and 20:00 is significantly lower than that in the other periods. In addition, it is also found that the bias is distinctly related with the geomagnetic latitude after we plot the auxiliary line of the geomagnetic latitude in dashed line. The bias values increased from the geomagnetic equator to the two geomagnetic poles, where the values in regions below 20° geomagnetic latitude can reach −15 TECU, and the values in regions above 40° geomagnetic latitude are close to 0 TECU. However, the biases near the sea at 20° south geomagnetic latitude seem to be anomaly, since the values obviously exceed 0 TECU. For COSMIC TEC, it is abnormal that the bias values are close to or greater than 0 TECU, because the orbital altitude of COSMIC satellites is far below that of GPS satellites.

The distribution of the scaling in Figure 8 has some similar characteristics to that in Figure 7, that is, there is also a clear numerical boundary around ±20° geomagnetic latitude during 08:00 to 24:00 (LT), and the scaling in these regions is maintained around 0.7~0.8, which lower than other regions. Moreover, there are same distribution of abnormal regions at the sea near 20° south geomagnetic latitude, and the scaling values in these regions exceed 1, specifically, the COSMIC TEC is greater than the IGS GIM TEC.

## 5. Summary and Conclusions

Due to the uneven distribution of global GNSS reference stations, the precision of IGS GIM products over oceanic areas is lower than that over continental areas. In this research, the precision of IGS GIM TEC was evaluated by comparing it with satellite-based SA and IRO TEC. The Jason-2 altimeter measurements from CNES and COSMIC measurements from CDAAC were utilized to retrieve the VTEC. The differences between IGS GIM and space-borne TEC are analyzed with biases and scaling factors modeled on different temporal and spatial environments. Both the biases and scaling factors show that the differences are variable with day time and night time, as well as with diurnal and latitude factors. The results show that the greater Jason-2 TEC values, the more the difference between Jason-2 and GIM TEC, for which the peak difference could reach around –10 TECU in low latitudes during the daytime. The scaling factors show a diurnal pattern with 0.7~0.9 at night and 0.9~1.0 during the daytime. For COSMIC products, the ΔTEC in the low latitudes of the northern hemisphere and near the equator is significantly higher than that in other regions, and the maximum difference could decrease to –20 TECU between 10:00 and 18:00 (LT) in all seasons. After 08:00 (LT), the distribution of the scaling begins to appear north-south symmetry with 0.85~0.95 around 40° latitudes and 0.75~0.8 around low latitudes.

The statistical results also show that in the southern hemisphere middle-high latitude area and some other central oceanic area, the space-borne TEC is higher than the IGS GIM one. Since the insufficient ground-based GNSS observations in such areas might result in a low precision of the extrapolated TEC values. Moreover, the approach of IGS GIM modeling by using a sun fixed coordinate might cause diurnal variations of incorrect modelling due to the boundary effect of the function model. As the precision of space-borne TEC should have even distribution around different areas of the Earth, it can be explained that the TEC over these areas are undervalued by the current GIM model, such that the space-borne SA and IRO techniques could be used as complementary observations to improve the accuracy and reliability of the TEC values in these areas. Furthermore, since the scaling factors are variable with season, local time and location, they could be applied as a given value but variable with temporal-spatial conditions while reconstructing TEC by means of multi-sensors. Therefore, the analysis of this research could provide some effective suggestions for TEC retrieval with combined GNSS, SA, and IRO observations.
